# Adaptor proteins regulating tumor-associated macrophage polarization during cancer progression

**DOI:** 10.18632/oncoscience.661

**Published:** 2026-05-22

**Authors:** Khandu Wadhonkar, Tomokazu Ohishi, Mirza S. Baig

**Affiliations:** ^1^Mehta Family School of Biosciences and Biomedical Engineering (MFS-BSBE), Indian Institute of Technology Indore (IITI), Indore, India; ^2^Institute of Microbial Chemistry (BIKAKEN), Laboratory of Oncology, Microbial Chemistry Research Foundation, 3-14-23 Kamiosaki, Shinagawa-ku, Tokyo 141-0021, Japan

**Keywords:** adaptor proteins, immunomodulation, tumor microenvironment, cancer, macrophage polarization

## Abstract

Adaptor proteins serve as essential molecular scaffolds within the tumor microenvironment, linking activated receptors to downstream signaling pathways and coordinating the assembly of multiprotein complexes that modulate immune responses. Emerging evidence suggests that adaptor proteins play a critical role in shaping macrophage phenotypic plasticity. Tumor-associated macrophages exhibit functional heterogeneity and can adopt either anti-tumorigenic phenotypes that promote immune activation or pro-tumorigenic phenotypes that support tumor growth, metastasis, and immune evasion. The dynamic transition between these functional states is tightly controlled by intracellular signaling networks in which adaptor proteins function as key regulatory nodes. Based on available mechanistic studies, we systematically summarize adaptor molecules that govern signaling pathways driving macrophage polarization within the cancer condition. Additionally, this review underscores the significance of adaptor proteins as key modulators of macrophage phenotype and highlights their potential as therapeutic targets for reprogramming macrophages to enhance anti-tumor immunity. Collectively, we provide a conceptual framework for understanding adaptor-mediated immune regulation in cancer and support the development of targeted strategies to shape the tumor microenvironment.

## INTRODUCTION

Cancer progression is strongly influenced by complex interactions between cancer cells and the surrounding immune microenvironment, where tumor-associated macrophages (TAMs) represent one of the most abundant and functionally adaptable immune cell populations [1]. In general, macrophages are present in distinct populations defined by both phenotypic markers and developmental origin. Monocyte-derived macrophages express high levels of CD11b, MHC class II, and CCR2 and originate from adult hematopoietic stem cells (HSC) via circulating monocytes [2]. On the other hand, tissue-resident macrophages are marked by elevated F4/80 and CX3CR1 expression and arise from embryonic progenitors, maintaining their populations predominantly through self-renewal within tissues [2]. Macrophages exhibit considerable plasticity that cannot be fully explained by the M1/M2 framework [3]. Advances in single-cell technologies, including single-cell RNA sequencing and mass cytometry, have transformed our understanding of macrophage heterogeneity [4]. This concept is reinforced by single-cell RNA-seq analysis from Xue et al., which uncovered 49 transcriptionally distinct macrophage states across 28 different activation stimuli [5]. Instead of a fixed polarization state, TAMs display adoptable functional behaviors and are continuously reshaped by tumor-derived clues, including soluble factors, extracellular vesicles (EVs), and damage-associated molecular patterns secreted by cancer cells [6–8]. In the tumor microenvironment (TME), macrophages undergo reprogramming and adopt a pro- or anti-tumor phenotype [9, 10]. Anti-tumoral type of macrophages can destroy tumor cells and prevent malignancy, whereas pro-tumoral type of macrophages support tumor growth by suppressing the immune response and facilitating angiogenesis and metastasis [11]. Macrophage responses to stimuli within TME are governed by intracellular signaling networks that link receptor activation to coordinated transcriptional regulation [12]. Within this framework, adaptor proteins facilitate a positive feedback loop within macrophages and cancer cells that drives tumor growth [13]. Dysregulation of these adaptor-mediated signaling has emerged as a crucial mechanism linking tumor cells signals to reprogram macrophages during cancer progression [14].

**Figure 1:**
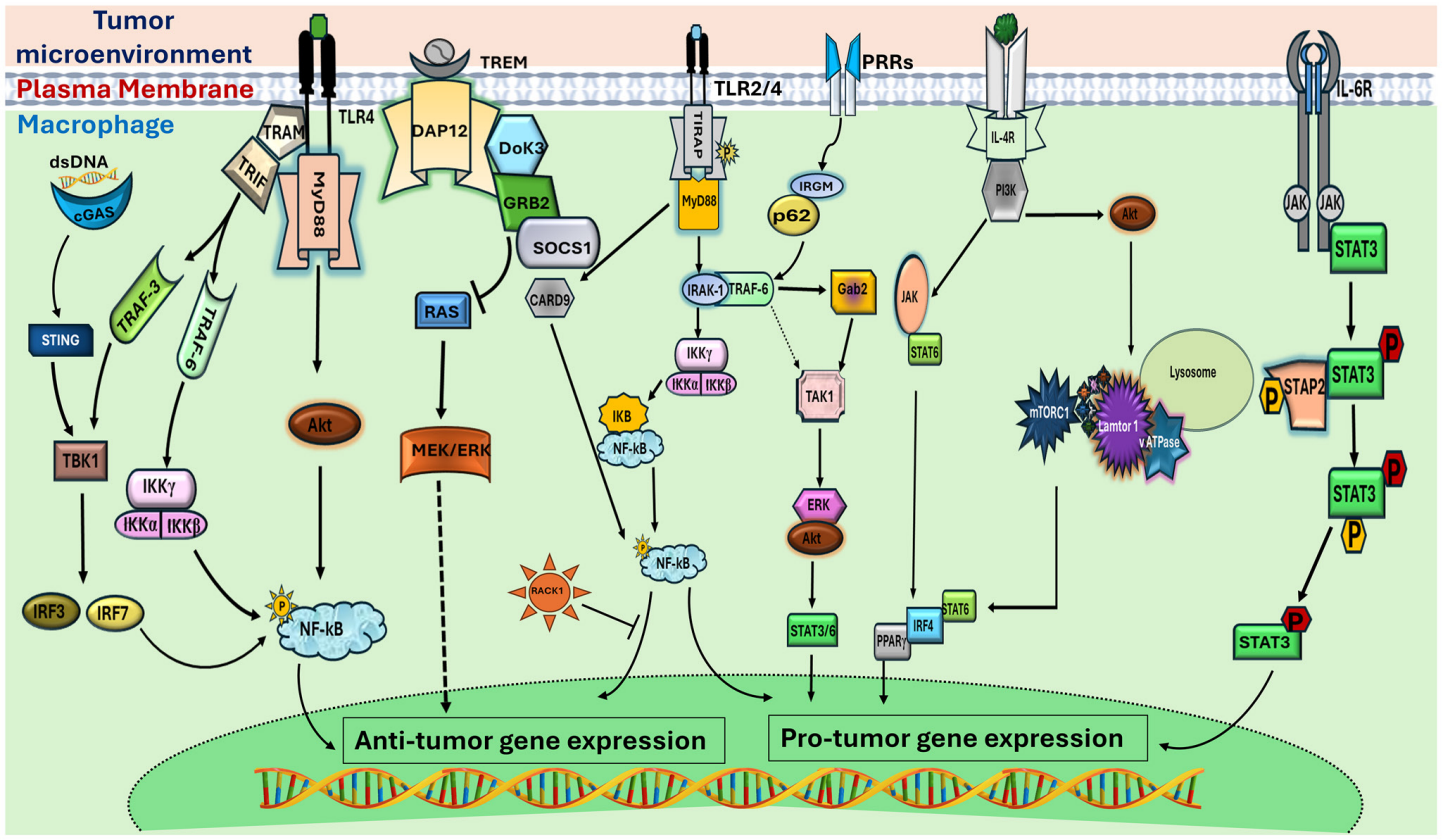
Adaptor protein–mediated signaling pathways regulating macrophage polarization within the tumor microenvironment. Schematic representation illustrating how adaptor proteins integrate receptor-mediated signaling pathways to affect macrophage functional states within the TME. Tumor-derived ligands and danger-associated molecular patterns activate multiple receptors on macrophages, including pattern recognition receptors (PRRs), TLR2/4, TREM, IL-4R, and IL-6R, etc. Upon receptor activation, adaptor proteins transmit signals to downstream pathways including NF-κB, TBK1–IRF3/7, MAPK (MEK/ERK), PI3K–Akt, mTORC1, and JAK–STAT signaling. Collectively, adaptor proteins act as critical molecular scaffolds that integrate signals from the TME to determine macrophage polarization toward anti-tumor or pro-tumor phenotypes, ultimately influencing tumor immunity and cancer progression.

Adaptor proteins represent an emerging class of proteins that play a significant role in these processes [15]. These adaptor molecules are a class of intracellular proteins characterized by two or more protein-binding domains and lack enzymatic activity, but can facilitate the formation of multi-protein complexes by binding with two or more proteins simultaneously [16]. These adaptor proteins act as molecular bridges, facilitating interactions between receptors and downstream signaling pathways to perform various cellular functions, including cell migration, proliferation, and differentiation [17]. Upon receptor activation, adaptor proteins transmit signals to downstream pathways including NF-κB, TBK1–IRF3/7, MAPK (MEK/ERK), PI3K–Akt, mTORC1, and JAK–STAT signaling [18, 19]. These adaptors serve as regulators in macrophage polarization, which in turn influences cancer progression, immune responses, and the TME via activation or inhibition of the inflammatory pathway [20]. Adaptor protein functions are highly interconnected, and their combined activity determines macrophage functional state within TME see in Figure 1. To understand the role of adaptor-mediated signaling in cancer conditions, adaptor proteins are categorized here based on their TAM functional states. The following are key adaptor molecules crucial in shaping the TME by influencing macrophage polarization, either promoting or inhibiting tumor progression and immune response.

## ADAPTORS LINKED TO PRO- AND ANTI-TUMOR MACROPHAGE STATES

Certain adaptor proteins involved in the transmission of signals and govern both pro-and anti- tumor macrophage function, influencing immune activation and tumor progression [21]. These adaptors operate conditionally in response to external stimuli and play a key role in shaping macrophages towards pro- and anti-tumor types within TME. MyD88-dependent signaling exhibits functional duality in TAMs and contributes to antitumor response under the acute phase, while supporting immunosuppressive response in the late phase [22, 23]. In TME, tumor-derived damage-associated molecular pattern (DAMP), such as HMGB1, heat shock proteins, extracellular nucleic acid and necrotic cell particles activate TLR/IL-R1-MyD88-MAPK/NF-κB signaling and contribute to tumor-associated signaling [23, 24]. Similarly, Nucleic acids or cyclic dinucleotide from stressed tumor cells activate TLR-TRIF or cGAS-STING signaling in macrophages, which trigger TBK1/IRF3-mediated response and can control tumor progression or inhibition [25, 26]. DAP12–SIRPβ complex recruits SYK and promotes phagocytosis [27–29], while TREM2-DAP12 signaling reshapes TAMs towards a tumor-supportive type [30]. In this section, we summarize major adaptor proteins that drive macrophage function towards tumor-promoting or tumor-restraining outcomes.

### Stimulator of interferon genes (STING)

STING serves as a central adaptor protein in innate immune response and activation, which is triggered by cytosolic nucleic acids derived from bacterial, viral, and damaged self-DNA in the cGAS–STING pathway [31]. Pan-cancer analysis demonstrates that STING protein expression is prevalent across multiple tumor types and correlates with aggressive cancer phenotypes [32]. cGAMP-activated STING serves as a signaling scaffold to recruit and activate TANK binding kinase 1 (TBK1), resulting in TBK1-mediated phosphorylation of STING at Ser366 [33]. This event promotes IRF3 activation and nuclear translocation, ultimately inducing interferon-stimulated genes and type I interferons [34]. Recent studies suggest that this signaling plays a context-dependent role in cancer, simultaneously promoting and restricting anti-tumor immune response across different stages of tumor immune interactions [34]. Lam KC et al. reported that microbiota-derived agonists activate type I interferon-derived signaling in intratumoral monocytes, facilitating macrophage polarization and reprogram TME towards antitumor response [35]. Similarly, Miao et al. showed that STING influences gastric cancer outcome with both its activation by 2′3′-cGAMP or knockdown and polarizes TAMs toward a pro-inflammatory state as well as induces apoptosis of cancer cells via IL6R–JAK–IL24 signaling axis [36]. This adaptor emerged as an interactive therapeutic target in cancer [37–39]. Preclinical studies show that STING agonists improve immune cells infiltration and antitumor immunity [40–42]. However, despite these promising results, most clinical trials of STING agonists have been discontinued or shown limited efficacy, and ongoing studies have yet to deliver the expected therapeutic benefits [43, 44]. These disappointing outcomes highlight the need to better understand context-dependent and alternative roles of STING signaling in cancer therapy.

### Myeloid differentiation factor 88 (MyD88)

The MyD88 acts as an adaptor protein and plays a pivotal role in the activation of innate immunity through the activation of immune cells via TLRs signaling. TLRs and MyD88 interaction are mainly associated with the activation of NF-κB and other signaling involved in inflammation [45]. MyD88 mainly elicits the immune response through the activation of NF-kB, AP-1, and IRFs transcription factors [46]. The influence of MyD88 on TME is crucial in cancer progression. MyD88- dependent pathways drive the expression of multiple genes involved in intestinal tumorigenesis and are required for both spontaneous and carcinogen-induced cancer development [22, 47]. Analysis of the glioma patient sample revealed significantly higher expression of MyD88 [46]. Breast cancer cells-derived exosomes initiate the proinflammatory molecules such as TNF-α, IL-6, CCL2, and GCSF in macrophages through the activation of TLR/MyD88/NF-kB signaling [48]. The alliance between TLR and MyD88 leads to recruitment and phosphorylation of IRAK family members, resulting in the activation of the TRAF6 adaptor which further activates TAK1 and subsequently activates IKK complex. Activated IKK phosphorylate IκB, upon phosphorylation, IκB undergoes degradation, which causes release as well as translocation of NF-κB into the nucleus and transcribes inflammatory genes as well as gene require for cell survival. Chen et al. reported in a high-fat diet-induced hepatocellular carcinoma (HCC) mouse model, MyD88 switches macrophages towards tumor supportive type via SREBP1/STAT6 signaling and enhanced non-alcoholic fatty liver disease-related HCC [49]. Interestingly, another study suggests that EMILIN-2, an extracellular matrix (ECM) molecule, triggers the activation of MyD88 dependent TLR-4/NF-κB signaling, promoting M1 macrophage population and affecting CRC development in a murine model [50]. Similarly, in the melanoma mouse model, MyD88/IL-1R signaling has been identified as a key regulator of PD-1 expression on tumor-associated macrophages, supporting their immunosuppressive phenotype [23]. The involvement of MyD88 in macrophage polarization during cancer progression has been reported in various studies. It represents a promising therapeutic target for enhancing macrophage-mediated anti-tumor immune responses.

### DNAX-activating protein of 12 kDa (DAP12)

DAP12, a 12 kDa immunoreceptor tyrosine-based activation motif (ITAM) containing transmembrane adaptor protein, expressed in cancer cells as well as in immune cells, including macrophages [30, 51]. DAP12 plays a functional role downstream of the TREM receptor family and plays a critical role in the regulation of immune response. Phosphorylated DAP12 triggers the activation of MAP/ERK signaling, and also controls the expression of inflammatory gene via regulation of NF-kB activation [52]. Interestingly DAP12 involvement is reported in SIRP-ß2 mediated signaling in myeloid cells. It recruited through binding of lysine residues present in transmembrane domain of SIRP-ß2 and promotes cancer cells phagocytosis, T cells activation and support innate anticancer immunity [53]. In the THP-1 monocyte, overexpression of SIRP-ß2 responsible for differentiation of monocytes towards macrophages [53]. Shabo et al. investigation reported that breast cancer patient tissue samples expressed DAP12 plays crucial role in macrophage fusion function and contributes to liver, bone metastasis and poor survival [30]. The study by Aoki et al. demonstrates that under leukemic conditions, the ITAM motif of DAP12 is essential for both macrophage differentiation and the maintenance of cell viability [29]. To target the DAP12 holds potential for improving immunotherapy outcomes in cancer-free survival.

### Toll-interleukin-1-receptor (TIR)-domain-containing adaptor-inducing interferon-β (TRIF)

TRIF is an adaptor protein recruited upon activation of TLR3 and TLR4 [54]. The activation of TRIF varies depending on cancer types, which can significantly impact immune response, either enhancing or inhibiting cancer [55, 56]. The expression has been detected in gastric cancer in mice and patient samples [57] and hepatocellular carcinoma patients [58]. TRIF is responsible for activating transcription factors such as interferon regulatory factor 3, nuclear factor kappa B, and activator protein, which contribute to the activation of interferon-β and production of various inflammatory cytokines [26, 59]. The interaction between TLR3 receptor and TRIF relies on the phosphorylation of tyrosine residues Tyr759 and Tyr858 within the cytoplasmic domain of the receptor [60, 61]. The determination of the downstream signaling axis is governed by the cytoplasmic domain of the receptor and its engagement with TRIF. In the production of interferon, TRIF binds with TRAF3 and facilitates the phosphorylation and activation of IRF3. Alongside, TRIF also interacts with TRAF6 and receptor-interacting protein 1 (RIP1) to activate NF-κB and drive inflammatory cytokine production [61]. Recently, Firmal P et al. reported that LPS stimulates the expression of SMAR1 via the TLR4-TRIF signaling axis, regulating STAT3 expression, and exerts antitumor effects by altering TAM towards the M1 phenotype of macrophages in the cell line of CRC [62]. The role of TRIF has also been investigated in apoptosis when overexpressed in 293T cells [63]. TRIF can be used as a potential target for immune modulation, contributing to the development of novel cancer immunotherapeutic strategies.

## ADAPTOR LINKED TO IMMUNOREGULATORY PRO-TUMOR MACROPHAGE STATE

The adaptor proteins mediated pro-tumoral macrophage reprogramming influenced by TME composition, including cancer cells-derived proteins as well as extracellular vesicles secreted in the surrounding [64, 65]. Within the TME, adaptor molecules participate in NF-κB, MAPK, PI3K-AKT, and IRF signaling pathways and alter macrophage fate towards tumor-supportive phenotypes. For example, growth factors and cytokines bind to their respective receptors, trigger phosphorylation, and recruit GRB2, which activates RAS-MAPK and PI3K-AKT signaling and governs macrophage polarization [66, 67]. TIRAP plays a functional role downstream of the TLR receptor and initiates NF-κB-dependent tumor-supportive state in macrophages [68]. In response to growth factor or G-protein-coupled receptor, the RIAM adaptor molecule is involved in PI3Kγ signaling and promotes immunoregulatory macrophage polarization [68, 69]. LAMTOR1, v-ATPase, mTORC1, and LXR-dependent gene expression support M2-like tumor supportive macrophage transition within TME [70]. Similarly, TRAF family adaptors are involved in TNF/IL1R/TLR signaling and promote tumor progression [71]. Cancer cells secreted VEGF, which activates SYK-CARD9-BCL-10-MALT1 signaling, promotes NF-κB mediated tumor supportive macrophage phenotype within TME [14]. Interesting finding suggested co-culture of THP-1 macrophages with oral squamous cell carcinoma-derived supernatant containing IL-1 and IL-6 activates respective receptors, recruits RACK1, and drives NF-κB signaling that promotes M2-like TAMs phenotype [72]. The STAP family receptor also acts downstream of the IL-6 receptor and STAP1/2-STAT3 signaling, promoting anti-inflammatory microglia in glioma [73]. Condition media from prostate cancer cells increases TRIB1 expression, resulting in suppression of IKB-zeta signaling, and leading to increased Bcl-3 and STAT3 activity, which promotes M2-like macrophage differentiation [74, 75]. Here, we provide a comprehensive overview of adaptor proteins that influence signaling pathways governing macrophage phenotypic switching toward tumor-supportive functions within the TME (see Figure 1).

### Grb2-associated binder2 (Gab2)

Gab2 belongs to the Gab family of proteins, which are characterized by their association with tyrosine kinases and the recruitment of phosphotyrosine-rich domain-containing molecules. These proteins play a crucial role in signaling mechanisms, significantly influencing cellular differentiation, proliferation, migration, and apoptosis [76]. The expression of Gab2 in different cancers has already been reported, such as in colorectal cancer patients’ tissue samples (CRC) [77], in the bone marrow and peripheral blood samples from chronic myeloid leukemia [78], breast cancer cells and cell line [79], and ovarian cancer patients [80], highlighting its role in tumor angiogenesis, metastasis, and progression. In the literature, it has been found that macrophages express Gab2, and the Gab2/SHP2/PI3K signaling pathway plays a key role in regulating cytokine secretion and phagocytosis in these cells [81]. Macrophages influence cancer progression through polarization shifts that change the balance of the TME [82]. Recently, the study done by Gao X et al. reported that CRC cells derived condition media treated TAM shows overexpression of Gab2, which is associated with poor prognosis [83]. In CRC patients, Gab2 polarizes the macrophages towards a tumor supportive state through the AKT and ERK signaling and promotes CRC growth as well as metastasis [83] The mechanism and influence of Gab2 on phenotype changes of TAMs remain unclear; the detailed mechanism of Gab2 control macrophage polarization provides further insight to develop new immunotherapeutic strategies targeting TAMs during cancer progression.

### Toll-interleukin-1 receptor (TIR) domain-containing adaptor protein (TIRAP)

The MyD88 adaptor-like (MAL)/TIRAP binds with MyD88 through the cytoplasmic TIR- domain and acts as bridge between Toll-like receptor 2/4 and its downstream signaling molecule to orchestrate the inflammatory response [84]. TIRAP can form homodimers or hetero dimers with MyD88 and activate AP-1 or NF-κB transcription factor-mediated proinflammatory response [85]. The expression of TIRAP has been reported in CRC cell lines [86], non-small cell lung cancer (NSCLC) [87], breast cancer cells [88], as well as in many other cancer types. In the coculture of LX2 and THP-1 condition, demethylation of TIRAP mRNA mediated by ALKBH5 controls the CCL5-CCR5 signaling pathway, which in turn promotes the recruitment of monocytes and M2 polarization of macrophages in liver fibrosis and hepatocellular carcinoma [89]. In macrophages, the TIRAP-mediated proinflammatory signaling axis binds with IRAK2, C-jun, as well as kinases like PKCδ and p38 MAPK [90]. The negative regulators SOCS1, CLIP170, IRAK1/4, and Triad3A are associated with ubiquitination and proteasomal degradation of TIRAP [84]. Despite TLR signaling remains persistently active in cancer, and the involvement of TIRAP in macrophage polarization is well established, its precise contribution to macrophage polarization within cancer remains unclear, underscoring a critical area for further investigation. Bridging this knowledge gap through targeted mechanistic investigations may help identify TIRAP-driven pathways as promising therapeutic opportunities for reprogramming tumor-associated macrophages.

### Rap1-interacting adaptor molecule (RIAM)

Rap1-interacting adaptor molecule is a crucial component in cellular responses and plays a crucial role during cell adhesion, migration, proliferation, and immune cells modulation in melanoma and prostate cancer [91]. RIAM belongs to the MRL (Mig-10/RIAM/Lamellipodin) family of adaptor proteins and functions as a mediator protein for Rap1 [92]. RIAM not only engages with activated Rap1 but also another signaling molecule through the Ras-association (RA) domain, a pleckstrin homology (PH) domain, and several proline-rich sequences [93]. Patsoukis N et al. reported RIAM integrates critical signaling pathways essential for the regulation of integrin-mediated immune responses and cancer progression [94]. In macrophage polarization, RIAM can direct reprogramming of macrophages towards tumor supportive phenotype and facilitate tumor progression, which has already been reported in the melanoma mice model [95]. RIAM plays a key role in the phagocytosis process by acting downstream of Rap1 and facilitating the engulfment of particles by macrophages. It achieves this by recruiting talin to complement receptors and rendering RIAM essential for complement-mediated phagocytosis in macrophages [95]. Overall, RIAM serves as a critical node for signal transduction in various immune cell processes and cancer progression.

### Late endosomal/lysosomal adaptor, MAPK and MTOR activator 1 (Lamtor1)

LAMTOR1 is an adaptor, connecting heterodimers, and is essential for the formation of regulator pentamer, which is required for mTORC1 activation during cellular signaling and organelle function [96]. LAMTOR1 can also act as a crucial anchor for p14-MP1–MEK–ERK axis within late endosomes and lysosomes, as well as activate RhoA and RhoC, and may shape actin remodelling [97]. Downregulation of LAMTOR1 has an impact on lysosomal activation and induces ROS-p53-dependent apoptosis [97]. The LAMTOR1 is essential in the proliferation of CD4+ T cells and the suppressive function of T regulatory cells [98]. The expression of LAMTOR1 has been reported in the cell lines of bladder cancer [99], non-small-cell lung cancer patient tissue [100], colon cancer mouse model [101] etc. LAMTOR1 undergoes LYS 63-linked polyubiquitination, which is influenced by TRAF4 ligase, which enhances regulators’ GEF activity, activates mTORC1, and influences inflammation-driven CRC [101]. The mTOR signaling influences the functional as well as metabolic differentiation of the pro- and anti-inflammatory polarization of macrophages within TME [102]. LAMTOR1 is crucial for immune-suppressive-type signaling by linking immune responses with metabolic processes [70]. Another study demonstrated that Lamtor1–/– macrophages, S6K phosphorylation is impaired, and 25-hydroxycholesterol levels are reduced, leading to altered LAMTOR1, v-ATPase, mTORC1, and LXR-dependent gene expression essential for M2 macrophage differentiation while simultaneously enhancing pro-inflammatory cytokine production in mice [96]. Researchers have already proposed Lamtor1 as a therapeutic target in cancer, primarily due to its role in promoting a tumor-supportive type of macrophage phenotype.

### Tumor necrosis factor receptor-associated factor (TRAF)

The TRAF family of proteins consists of cytoplasmic adaptor proteins that bind directly with the intracellular domains of cell surface receptors and was initially identified through their association with members of the TNF receptor superfamily [103]. In mammals, the TRAF family currently comprises six classical members (TRAF1-TRAF6) that possess a conserved TRAF domain at the carboxy terminus, and one nonclassical member (TRAF7) that lacks this TRAF domain [71]. The involvement of the TRAF family adaptor has been found in cell proliferation, differentiation, survival, and apoptosis, as well as in immune modulation [71]. In mouse models, TRAF1 and TRAF4 influence skin and lung carcinoma [104–106]. TRAF2 promotes tumor development in breast [107] and gastric cancer in mice and patient samples, respectively [108]. TRAF5 contributes to the development of CRC within human patients [109]. TRAF6 is upregulated in several tumor types, including colorectal, gastric, breast cancer, and is specifically involved in binding with TNF receptor as well as IL1R/TLR signaling [71]. Tan et al. reported, baicalin mediates the autophagic degradation of TRAF2 in TAMs, thereby modulating TRAF2/TRAF3 interactions with IKKα/RelB/p52 signaling axis [110]. This indirect activation of the RelB/p52 signaling pathway upregulates CXCL12 and CCL9 expression, leading to overexpression of IL-6 and TNF-α, and promotes the polarization of TAM from anti-tumor to tumor-supportive types in the orthotopic HCC implantation mice model [110]. In CRC, an increase in M1 macrophage populations was detected after TRAF6 knockdown in a mouse model [111]. In TME, TRAF2 modulates the production of inflammatory cytokines in TAMs and promotes tumor progression [112]. Elucidating more mechanistic insights into TRAF family adaptor-mediated signaling will open new opportunities for developing effective therapeutic interventions in cancer.

### Caspase-recruitment domain-containing adaptor protein (CARD9)

CARD9 adaptor is a crucial regulator involved in innate and adaptive immunity and modulates inflammatory responses and oxidative stress by controlling the expression as well as production of key cytokines and chemokines [113]. CARD9 facilitates tumor cell proliferation and migration, and contributes to cancer development [114] and is expressed in lung adenocarcinoma patients [115], ovarian cancer tissue and cell lines [116], CRC [117]. In the colitis-associated cancer mice model, CARD9 signaling axis drives IL-1β production in the damaged intestine, which stimulates IL-22 secretion in lymphoid cells, leading to STAT3 activation and subsequent tumorigenesis in transformed epithelium [118]. The overexpression of CARD9 is reported in the infiltrated macrophages, which results in increased secretion of tumor growth-supporting cytokines (IL-10 and IL-1α) while reduced levels of the tumor growth-inhibiting cytokine IL-12, and contributes to tumor metastasis during colon cancer progression [14]. In human CRC progression, upregulated expression of CARD9 in infiltrating macrophages has been observed [14]. Mechanistically, CARD9 changes the phenotype of macrophages towards metastasis-promoting type through the activation of the NF-κB signaling pathway [14]. The involvement of CARD9 in tumor cell proliferation and metastasis emphasizes its potential as a therapeutic target in anti-cancer strategies.

### Signal-transducing adaptor protein (STAP)

The signal-transducing adaptor protein (STAP) family comprises two adaptor molecules STAP-1 and STAP-2, which contain a Pleckstrin homology (PH) domain in their N-terminal region and contribute to multiple signaling cascades [119]. The expression of STAP-1 is upregulated in chronic myeloid leukemia (CML) stem cells [120], while STAP-2 is expressed in a variety of immune cell types and tissues [121]. Studies reported that in the orthotopic mice model, expression of STAP1 in glioma-associated microglia positively correlates with tumor aggressiveness as well as poor prognosis in glioma and potentially facilitates M2-like polarization through IL-6/STAT3 signaling and inhibiting phagocytosis [73]. STAP-2 mediates Brk-dependent phosphorylation and activation of STAT3 and STAT5 to promote breast cancer cells proliferation [122]. STAP-2 is highly expressed in prostate cancer and upregulates the epidermal growth factor receptor (EGFR) signaling [123]. STAP-2 also serves as a critical regulator of both innate and adaptive immune responses by engaging in cytokine signaling axis through its interactions with STAT family transcription factors [124]. In Raw macrophages, STAP-2 directly promotes LPS-induced activation of the NF-κB signaling pathway through its interaction with MyD88 and IKK-αβ and serves as a novel adaptor protein that links MyD88 and IKK-αβ in TLR4 signaling [125]. The involvement of these adaptors in macrophage polarization during malignancy could offer valuable insights into their potential therapeutic applications in cancer treatment.

### Receptor for activated C kinase 1 (RACK1)

Receptor for activated C kinase 1 (RACK1) belonging to the tryptophan-aspartate repeat (WDR) scaffold protein family and identified as an interacting partner of activated PKC kinase [126]. RACK1 has been engages with receptor proteins as well as protein kinases and play a role in various biological processes, including cell migration [127], angiogenesis [128], and cancer metastasis [129]. RACK1 is widely expressed in normal tissues and is significantly upregulated in different kinds of cancer and is believed to contribute to the development and progression of cancer [130]. The upregulated RACK1 expression positively correlates with tumor differentiation and lymph node metastasis in colon cancer, while negatively associated with patient survival [130]. RACK1 also promotes tumorigenicity in non-small cell lung cancer [131], oral squamous cell carcinoma (OSCC) [132], pulmonary adenocarcinomas [133], and breast cancer [134]. The study conducted by Dan et al. demonstrated that in a clinical sample of patients, RACK1 can inhibit the activation of nuclear factor-kappa B (NF-κB), modulate the expression of IL-6, CCL5, and CSF released by tumor cells, and attenuate prolonged inflammatory responses in oral squamous cell carcinoma (OSCC) [72]. The upregulated RACK1 expression in OSCC inhibits macrophage activation but promotes an increased proportion of M2/M1 ratio *in vitro* as well as *in vivo* and fosters malignancy [72]. By regulating crucial signaling mechanisms, RACK1 contributes to cancer progression and could be considered a potential therapeutic target for cancer treatment.

### Tribbles 1(TRIB 1)

TRIB1 (Tribbles homolog 1) belongs to the evolutionarily conserved mammalian tribbles homolog pseudokinase family and serves as an adaptor instead of direct phosphorylation of the target molecule [135]. The lack of a functional adenosine 5’-triphosphate (ATP) binding site serves as a scaffold and facilitates the assembly of other proteins [136]. The expression of TRIB1 exhibits a positive correlation with the levels of nuclear factor NF-κB and interleukin-8 in breast cancer patients [74, 137]. IL-8 is involved in the activation of the surface receptors CXCR1 and CXCR2 expressed by TAM and induces to secrete more growth factors [138]. In the murine breast cancer model, TRIB1 controls cancer proliferation by modulating the phenotypes of TAMs [139]. In the mouse model, TRIB1 influences cytokine secretion by inhibiting IκB-zeta in prostate cancer cells, which promotes the differentiation of monocytes into M2 macrophages and facilitates prostate cancer progression [74]. TRIB1 interactions with various cellular signaling molecules suggest its potential as a biomarker for cancer prognosis and as a target for novel therapeutic strategies.

### p62 (SQSTM1)

p62 is a conserved, multifunctional adaptor molecule involved in many important cellular processes, and upregulated expression was found in many cancer cells, which supports cancer progression [140, 141]. Because of its distinct domain architecture, p62 binds with multiple binding partners and modulates pathways controlling tissue homeostasis, inflammation, and cancer [142]. p62 positively increases NF-κB activity at the molecular level and contributes to inflammation and cancer progression [142]. P62 also contributes to host metabolic control by altering the activity of immune cells within the TME [143]. It enhances lactate production in cancer cells by regulating glucose metabolism, promoting TME acidification, leading to changing macrophage phenotype towards an M2-like state, and contributing to tumor evasion [143]. In the glioma mouse model, immunity-related GTPase M (IRGM) switches macrophages towards M2-like macrophage phenotype via the p62/TRAF6/NF-κB pathway and promotes glioma progression [144]. In another interesting study, it was found that silencing of p62 diminishes ox-LDL-induced proinflammatory response in macrophages by inhibition of mTOR/NF-κB signaling pathways [145]. More mechanistic insights into p62-mediated inflammation and cancer progression may guide the discovery of new therapeutic interventions. The development of small-molecule agents against p62 has been identified as a promising therapeutic avenue in hepatocellular carcinoma management [143].

## IMPACT OF ADAPTOR MOLECULE ON CANCER PROGRESSION AND METASTASIS

Following the discussion of individual adaptor proteins in TAM polarization, we now examine their roles in cancer progression, metastasis, and immune evasion. For example, the GRB2 adaptor is expressed in different cancers and serves an important role in tumor angiogenesis, metastasis as well as progression through the activation of AKT and ERK signaling [83]. Breast cancer cells expressing DAP12 show an impact on macrophage functions and contribute to liver, bone metastasis and poor survival [30]. The adaptor, like MYD88, TIRAP, and TRIF activated through TLR signaling and significantly contributes to TME and macrophage polarization [48, 62, 89]. RIAM can influence the reprogramming of macrophages into a tumor-promoting M2 phenotype, which aids in tumor progression [95]. LAMTOR1 mediates the polarization of macrophages toward the M2 phenotype [70]. TRAF family proteins also govern macrophage polarization according to their interactions, leading to either pro-inflammatory or anti-inflammatory responses within the TME [110, 146]. In CRC, CARD9 promotes tumor growth by enhancing the expression of anti-inflammatory cytokines such as IL-10 and IL-1α, while simultaneously reducing the expression of IL-12 in tumor-infiltrating macrophages [14]. The STAP1 expression in glioma-associated microglia correlates with tumor aggressiveness and inhibits phagocytosis by promoting M2-like polarization through IL-6/STAT3 signaling [73]. In OSCC overexpression of RACK-1 increases the proportion of M2/M1 ratio of macrophages and contributes to malignancy [72]. The involvement of adaptor proteins in tumor proliferation and metastasis highlights their potential as therapeutic targets in anti-cancer strategies.

## THERAPEUTIC STRATEGIES TARGETING ADAPTOR MOLECULES

Therapeutic strategies targeting adaptor molecules offer a promising approach in the fight against a range of diseases, including cancer [147]. Adaptor molecules play critical roles in cellular signaling pathways that govern tumor growth, metastasis, and immune responses [21]. Targeting adaptor protein interactions using small molecules or peptides represents a potential strategy to alter TAM response in cancer [148, 149]. Gene-silencing approaches such as siRNA or CRISPR/Cas9 can downregulate adaptor expression, while interfering with receptor–adaptor binding or modulating post-translational modifications may further impair pro-tumoral macrophage activity. Lin Xie et al. reported that the synthetic compound TJ-M2010-5 targets MyD88 homodimerization and effectively prevents colitis-associated CRC [150]. Another investigation reported that ST2825, a MyD88 inhibitor, effectively suppresses the activation of the NF-κB/AKT1/p21 signaling axis and increases cell cycle arrest and apoptosis in pancreatic cancer cells [151]. Ewa Witort et al. employed a combination of antisense oligonucleotides and a proteasome inhibitor targeting the tumor necrosis factor receptor type 1-associated death domain (TRADD) adaptor to treat chemoresistant hepatocellular carcinoma cells [152]. Adaptor proteins have gained significant attention recently because of their crucial role in cancer. Several clinical trials have shown promising results by targeting TAMs in various cancer types [153, 154]; however, these approaches have not specifically focused on adaptor proteins. Although preclinical studies highlight the role of adaptor proteins in macrophage polarization and cancer progression, no clinical trials or approved therapies currently target these molecules in macrophages. This remains an important and emerging area for future investigation. Despite increasing evidence linking adaptor proteins to macrophage polarization and tumor progression, their therapeutic targeting remains challenging. Adaptor proteins function within complex and interconnected signaling networks, where redundancy and compensatory pathways allow cancer and immune cells to bypass the inhibition of individual adaptors [155, 156]. In addition, many adaptor proteins participate in multiple physiological pathways, raising concerns about specificity and potential systemic effects. Therefore, a deeper understanding of their context-dependent functions and interactions within the TME is required before adaptor-targeted strategies can be effectively translated into cancer immunotherapy.

## CONCLUSIONS

Adaptor proteins are essential for regulating TAM polarization during cancer progression. These molecules serve as molecular bridges, promoting interactions between cell-surface receptors and downstream signaling molecules within the TME, and they influence cancer growth and metastasis. Recent studies have emphasized the critical role of adaptor proteins in macrophage reprogramming see Table 1. Collectively, these adaptor proteins can serve as potential therapeutic and diagnostic targets in cancer. Investigating the role of adaptor proteins in this context could lead to the development of novel therapeutic strategies targeting macrophage plasticity in cancer treatment and to innovative strategies for reprogramming TAMs toward anti-tumor phenotypes, ultimately improving outcomes in cancer treatment. Although recent advances have elucidated the roles of several adaptor proteins in TAM polarization, the majority of studies remain at the preclinical stage. Moreover, the context-dependent dual functions of certain adaptors (e.g., STING, MyD88, DAP12) in promoting both anti- and pro-tumor phenotypes underscore the complexity of adaptor-mediated signaling within the TME. Future investigations should focus on clarifying cell-type-specific adaptor functions using single-cell transcriptomics [157], developing highly selective small-molecule inhibitors or PROTACs that target critical adaptor–receptor interactions [158], and exploring combination strategies involving immune checkpoint blockade or TAM-depleting agents [159, 160]. Considering the crucial role of adaptor proteins in signal transduction [161], we can expect significant advancements in this area of research in the future. Such progress will enhance our appreciation and understanding of adaptor proteins in signal transduction and their potential as therapeutic targets.

**Table 1:** Adaptor proteins involved in regulating macrophage polarization within the tumor microenvironment (TME) and their roles in cancer progression

S. No	Adaptor protein	Receptor/Stimulus	Major signaling	Role in cancer progression	Key references
**Adaptors linked to pro- and anti-tumor macrophage states**					
1.	***STING***	Cytosolic DNA	cGAS-STING signaling	TBK1/IRF3 signaling drives context-dependent macrophage polarization, modulating anti- or pro-tumor immune responses.	[25, 36]
2.	***MyD88***	TLRs, IL-1R	TLR/ILR1-MyD88-MAPK/NF-κB signaling	Promotes anti-tumor macrophage polarization initially, but over time drives pro-tumor TAM polarization and metastasis	[22–24]
3.	***DAP12***	SIRPβ, TREM2	DAP12–SIRPβ, TREM2-DAP12 signaling	DAP12–SIRPβ–SYK enhances macrophage phagocytosis, whereas TREM2–DAP12 signaling promotes tumor-supportive TAM polarization	[27–30]
4.	***TRIF***	LPS	TLR-NF-κB signaling	Reprograms TAMs toward an anti-tumor phenotype via STAT3 inhibition, suppressing tumor growth	[26]
**Adaptor linked to immunoregulatory pro-tumor macrophage state**					
5.	**Gab2**	Growth factors and cytokines	RAS-MAPK and PI3K-AKT signaling	Promotes pro-tumoral M2-like TAM polarization in the TME, enhancing cancer cell proliferation and survival	[66, 67]
6.	**TIRAP**	Activation of TLR2/4 by tumor-derived ligands	TLR2/4–MyD88–NF-κB/MAPK signaling	Drives tumor-supportive TAM polarization, promoting immunoregulation and tumor cell proliferation.	[68, 89]
7.	**RIAM**	Growth factor or G-protein-coupled receptor	Erk1/2 MAPK and PI3Kγ signaling	Regulates integrin-mediated immune responses and macrophage adhesion	[68, 69, 91, 95]
8.	**LAMTOR1**	IL-4/IL-4R	p14-MP1–MEK–ERK axis, mTORC1 pathway	Drives M2-like macrophage polarization via LAMTOR1–mTORC1–LXR signaling and RhoA/RhoC-mediated actin remodelling	[70, 101]
9.	***TRAF***	TNF, IL-1	TNF/IL1R/TLR signaling, NF-κB signaling	Promotes M2-like macrophage polarization, facilitating inflammation-driven tumor progression	[71]
10.	**CARD9**	VEGF	SYK-CARD9-BCL-10-MALT1 -NF-κB signaling	Promotes metastasis-associated macrophage gene expression and tumor-promoting cytokine production	[14, 162]
11.	***STAP***	IL-6 receptor	STAP1/2-STAT3 signaling	Skews macrophages toward a pro-tumoral phenotype, enhancing immunosuppression and tumor growth	[73]
12.	***RACK1***	IL-1, IL-6	IL-1R/IL-6R RACK1 activated NF-κB signaling	Regulates macrophage M2/M1 balance and immunoregulatory tone of the TME	[72]
13.	**TRIB1**	CXCL2, IL-8	IκB-zeta signaling	Promotes monocyte-to–M2-like macrophage differentiation and cytokine secretion via IκB-ζ inhibition, supporting tumor proliferation	[74, 136, 139]
14.	**p62 (SQSTM1)**	IL8, CXCR1/CXCR2 Receptor	p62/TRAF6/NF-κB pathway	Induces tumor-supportive macrophage phenotype and supports tumor-promoting inflammation	[144]

## References

[1] Pittet MJ, Michielin O, Migliorini D. Clinical relevance of tumour-associated macrophages. Nat Rev Clin Oncol. 2022; 19:402–21. 10.1038/s41571-022-00620-6. 35354979

[2] Mass E, Ballesteros I, Farlik M, Halbritter F, Günther P, Crozet L, Jacome-Galarza CE, Händler K, Klughammer J, Kobayashi Y, Gomez-Perdiguero E, Schultze JL, Beyer M, et al. Specification of tissue-resident macrophages during organogenesis. Science. 2016; 353:aaf4238. 10.1126/science.aaf4238. 27492475 PMC5066309

[3] Zhao W, Zhang Z, Xie M, Ding F, Zheng X, Sun S, Du J. Exploring tumor-associated macrophages in glioblastoma: from diversity to therapy. Npj Precis Oncol. 2025; 9:126. 10.1038/s41698-025-00920-x. 40316746 PMC12048723

[4] Yan L, Wang J, Cai X, Liou Y, Shen H, Hao J, Huang C, Luo G, He W. Macrophage plasticity: signaling pathways, tissue repair, and regeneration. MedComm. 2024; 5:e658. 10.1002/mco2.658. 39092292 PMC11292402

[5] Xue J, Schmidt SV, Sander J, Draffehn A, Krebs W, Quester I, De Nardo D, Gohel TD, Emde M, Schmidleithner L, Ganesan H, Nino-Castro A, Mallmann MR, et al. Transcriptome-based network analysis reveals a spectrum model of human macrophage activation. Immunity. 2014; 40:274–88. 10.1016/j.immuni.2014.01.006. 24530056 PMC3991396

[6] Xu J, Ding L, Mei J, Hu Y, Kong X, Dai S, Bu T, Xiao Q, Ding K. Dual roles and therapeutic targeting of tumor-associated macrophages in tumor microenvironments. Signal Transduct Target Ther. 2025; 10:268. 10.1038/s41392-025-02325-5. 40850976 PMC12375796

[7] Wadhonkar K, Das D, Kant Chittela R, Obukhov AG, Baig MS. Role of colorectal cancer-derived exosomes in modulating macrophage phenotype during tumor development. Carcinogenesis. 2025; 47:bgag003. 10.1093/carcin/bgag003. 41554687

[8] Zhao J, Wang B, Li X, Wei C, Min Y, Wang D. Single-cell transcriptomic analysis reveals the heterogeneity and functional characteristics of macrophage subpopulations in colon cancer. Discov Oncol. 2025; 17:101. 10.1007/s12672-025-04002-z. 41276730 PMC12816490

[9] Wynn TA, Chawla A, Pollard JW. Macrophage biology in development, homeostasis and disease. Nature. 2013; 496:445–55. 10.1038/nature12034. 23619691 PMC3725458

[10] Wadhonkar K, Singh N, Heralde FM, Parihar SP, Hirani N, Baig MS. Exosome-derived miRNAs regulate macrophage-colorectal cancer cell cross-talk during aggressive tumor development. Colorectal Cancer. 2023; 12:CRC40. 10.2217/crc-2022-0012.

[11] Gordon S. Alternative activation of macrophages. Nat Rev Immunol. 2003; 3:23–35. 10.1038/nri978. 12511873

[12] Li M, Wang M, Wen Y, Zhang H, Zhao G, Gao Q. Signaling pathways in macrophages: molecular mechanisms and therapeutic targets. MedComm. 2023; 4:e349. 10.1002/mco2.349. 37706196 PMC10495745

[13] Xu Y, Li L, Yang W, Zhang K, Zhang Z, Yu C, Qiu J, Cai L, Gong Y, Zhang Z, Zhou J, Gong K. TRAF2 promotes M2-polarized tumor-associated macrophage infiltration, angiogenesis and cancer progression by inhibiting autophagy in clear cell renal cell carcinoma. J Exp Clin Cancer Res. 2023; 42:159. 10.1186/s13046-023-02742-w. 37415241 PMC10324183

[14] Yang M, Shao JH, Miao YJ, Cui W, Qi YF, Han JH, Lin X, Du J. Tumor cell-activated CARD9 signaling contributes to metastasis-associated macrophage polarization. Cell Death Differ. 2014; 21:1290–302. 10.1038/cdd.2014.45. 24722209 PMC4085533

[15] Flynn DC. Adaptor proteins. Oncogene. 2001; 20:6270–72. 10.1038/sj.onc.1204769. 11607828

[16] Borowicz P, Chan H, Hauge A, Spurkland A. Adaptor proteins: Flexible and dynamic modulators of immune cell signaling. Scand J Immunol. 2020; 92:e12951. 10.1111/sji.12951. 32734639

[17] Chen Y, Gu Y, Xiong X, Zheng Y, Liu X, Wang W, Meng G. Roles of the adaptor protein tumor necrosis factor receptor type 1-associated death domain protein (TRADD) in human diseases. Biomed Pharmacother. 2022; 153:113467. 10.1016/j.biopha.2022.113467. 36076575

[18] Lee CC, Avalos AM, Ploegh HL. Accessory molecules for Toll-like receptors and their function. Nat Rev Immunol. 2012; 12:168–79. 10.1038/nri3151. 22301850 PMC3677579

[19] Kawasaki T, Kawai T. Toll-like receptor signaling pathways. Front Immunol. 2014; 5:461. 10.3389/fimmu.2014.00461. 25309543 PMC4174766

[20] Kerneur C, Cano CE, Olive D. Major pathways involved in macrophage polarization in cancer. Front Immunol. 2022; 13:1026954. 10.3389/fimmu.2022.1026954. 36325334 PMC9618889

[21] Baig MS, Barmpoutsi S, Bharti S, Weigert A, Hirani N, Atre R, Khabiya R, Sharma R, Sarup S, Savai R. Adaptor molecules mediate negative regulation of macrophage inflammatory pathways: a closer look. Front Immunol. 2024; 15:1355012. 10.3389/fimmu.2024.1355012. 38482001 PMC10933033

[22] Salcedo R, Cataisson C, Hasan U, Yuspa SH, Trinchieri G. MyD88 and its divergent toll in carcinogenesis. Trends Immunol. 2013; 34:379–89. 10.1016/j.it.2013.03.008. 23660392 PMC3847901

[23] Tartey S, Neale G, Vogel P, Malireddi RKS, Kanneganti TD. A MyD88/IL1R Axis Regulates PD-1 Expression on Tumor-Associated Macrophages and Sustains Their Immunosuppressive Function in Melanoma. Cancer Res. 2021; 81:2358–72. 10.1158/0008-5472.CAN-20-3510. 33619117 PMC11645125

[24] Yu L, Wang L, Chen S. Endogenous toll-like receptor ligands and their biological significance. J Cell Mol Med. 2010; 14:2592–603. 10.1111/j.1582-4934.2010.01127.x. 20629986 PMC4373479

[25] Corrales L, Glickman LH, McWhirter SM, Kanne DB, Sivick KE, Katibah GE, Woo SR, Lemmens E, Banda T, Leong JJ, Metchette K, Dubensky TW Jr, Gajewski TF. Direct Activation of STING in the Tumor Microenvironment Leads to Potent and Systemic Tumor Regression and Immunity. Cell Rep. 2015; 11:1018–30. 10.1016/j.celrep.2015.04.031. 25959818 PMC4440852

[26] Yamamoto M, Sato S, Hemmi H, Hoshino K, Kaisho T, Sanjo H, Takeuchi O, Sugiyama M, Okabe M, Takeda K, Akira S. Role of Adaptor TRIF in the MyD88-Independent Toll-Like Receptor Signaling Pathway. Science. 2003; 301:640–43. 10.1126/science.1087262. 12855817

[27] Hayashi A, Ohnishi H, Okazawa H, Nakazawa S, Ikeda H, Motegi S, Aoki N, Kimura S, Mikuni M, Matozaki T. Positive Regulation of Phagocytosis by SIRPβ and Its Signaling Mechanism in Macrophages. J Biol Chem. 2004; 279:29450–60. 10.1074/jbc.M400950200. 15123631

[28] Molgora M, Esaulova E, Vermi W, Hou J, Chen Y, Luo J, Brioschi S, Bugatti M, Omodei AS, Ricci B, Fronick C, Panda SK, Takeuchi Y, et al. TREM2 Modulation Remodels the Tumor Myeloid Landscape Enhancing Anti-PD-1 Immunotherapy. Cell. 2020; 182:886–900.e17. 10.1016/j.cell.2020.07.013. 32783918 PMC7485282

[29] Aoki N, Kimura S, Oikawa K, Nochi H, Atsuta Y, Kobayashi H, Sato K, Katagiri M. DAP12 ITAM Motif Regulates Differentiation and Apoptosis in M1 Leukemia Cells. Biochem Biophys Res Commun. 2002; 291:296–304. 10.1006/bbrc.2002.6434. 11846404

[30] Shabo I, Olsson H, Stål O, Svanvik J. Breast Cancer Expression of DAP12 is Associated With Skeletal and Liver Metastases and Poor Survival. Clin Breast Cancer. 2013; 13:371–77. 10.1016/j.clbc.2013.05.003. 23810293

[31] Sun X, Ni Y, He Y, Yang M, Tani T, Kitajima S, Barbie DA, Li J. Engineering the Immune Adaptor Protein STING as a Functional Carrier. Adv Ther. 2021; 4:2100066. 10.1002/adtp.202100066.

[32] Menz A, Zerneke J, Viehweger F, Büyücek S, Dum D, Schlichter R, Hinsch A, Bawahab AA, Fraune C, Bernreuther C, Kluth M, Hube-Magg C, Möller K, et al. Stimulator of Interferon Genes Protein (STING) Expression in Cancer Cells: A Tissue Microarray Study Evaluating More than 18,000 Tumors from 139 Different Tumor Entities. Cancers. 2024; 16:2425. 10.3390/cancers16132425. 39001487 PMC11240524

[33] Zhang C, Shang G, Gui X, Zhang X, Bai X, Chen ZJ. Structural basis of STING binding with and phosphorylation by TBK1. Nature. 2019; 567:394–98. 10.1038/s41586-019-1000-2. 30842653 PMC6862768

[34] Zhang X, Chen Y, Liu X, Li G, Zhang S, Zhang Q, Cui Z, Qin M, Simon HU, Terzić J, Kocic G, Polić B, Yin C, et al. STING in cancer immunoediting: Modeling tumor-immune dynamics throughout cancer development. Cancer Lett. 2025; 612:217410. 10.1016/j.canlet.2024.217410. 39826670

[35] Lam KC, Araya RE, Huang A, Chen Q, Di Modica M, Rodrigues RR, Lopès A, Johnson SB, Schwarz B, Bohrnsen E, Cogdill AP, Bosio CM, Wargo JA, et al. Microbiota triggers STING-type I IFN-dependent monocyte reprogramming of the tumor microenvironment. Cell. 2021; 184:5338–56.e21. 10.1016/j.cell.2021.09.019. 34624222 PMC8650838

[36] Miao L, Qi J, Zhao Q, Wu QN, Wei DL, Wei XL, Liu J, Chen J, Zeng ZL, Ju HQ, Luo H, Xu RH. Targeting the STING pathway in tumor-associated macrophages regulates innate immune sensing of gastric cancer cells. Theranostics. 2020; 10:498–515. 10.7150/thno.37745. 31903134 PMC6929973

[37] Haag SM, Gulen MF, Reymond L, Gibelin A, Abrami L, Decout A, Heymann M, Van Der Goot FG, Turcatti G, Behrendt R, Ablasser A. Targeting STING with covalent small-molecule inhibitors. Nature. 2018; 559:269–73. 10.1038/s41586-018-0287-8. 29973723

[38] Wang Y, Luo J, Alu A, Han X, Wei Y, Wei X. cGAS-STING pathway in cancer biotherapy. Mol Cancer. 2020; 19:136. 10.1186/s12943-020-01247-w. 32887628 PMC7472700

[39] Decout A, Katz JD, Venkatraman S, Ablasser A. The cGAS–STING pathway as a therapeutic target in inflammatory diseases. Nat Rev Immunol. 2021; 21:548–69. 10.1038/s41577-021-00524-z. 33833439 PMC8029610

[40] Hines JB, Kacew AJ, Sweis RF. The Development of STING Agonists and Emerging Results as a Cancer Immunotherapy. Curr Oncol Rep. 2023; 25:189–99. 10.1007/s11912-023-01361-0. 36705879 PMC10994474

[41] Jiang M, Jia K, Wang L, Li W, Chen B, Liu Y, Wang H, Zhao S, He Y, Zhou C. Alterations of DNA damage response pathway: Biomarker and therapeutic strategy for cancer immunotherapy. Acta Pharm Sin B. 2021; 11:2983–94. 10.1016/j.apsb.2021.01.003. 34729299 PMC8546664

[42] Amouzegar A, Chelvanambi M, Filderman J, Storkus W, Luke J. STING Agonists as Cancer Therapeutics. Cancers. 2021; 13:2695. 10.3390/cancers13112695. 34070756 PMC8198217

[43] Li Y, Li X, Yi J, Cao Y, Qin Z, Zhong Z, Yang W. Nanoparticle-Mediated STING Activation for Cancer Immunotherapy. Adv Healthc Mater. 2023; 12:2300260. 10.1002/adhm.202300260. 36905358

[44] Sallets A, Robinson S, Kardosh A, Levy R. Enhancing immunotherapy of STING agonist for lymphoma in preclinical models. Blood Adv. 2018; 2:2230–41. 10.1182/bloodadvances.2018020040. 30194137 PMC6134215

[45] Zheng H, Wu X, Guo L, Liu J. MyD88 signaling pathways: role in breast cancer. Front Oncol. 2024; 14:1336696. 10.3389/fonc.2024.1336696. 38347830 PMC10859757

[46] Guo Q, Xiao X, Zhang J. MYD88 Is a Potential Prognostic Gene and Immune Signature of Tumor Microenvironment for Gliomas. Front Oncol. 2021; 11:654388. 10.3389/fonc.2021.654388. 33898320 PMC8059377

[47] Rakoff-Nahoum S, Medzhitov R. Regulation of Spontaneous Intestinal Tumorigenesis Through the Adaptor Protein MyD88. Science. 2007; 317:124–27. 10.1126/science.1140488. 17615359

[48] Chow A, Zhou W, Liu L, Fong MY, Champer J, Van Haute D, Chin AR, Ren X, Gugiu BG, Meng Z, Huang W, Ngo V, Kortylewski M, Wang SE. Macrophage immunomodulation by breast cancer-derived exosomes requires Toll-like receptor 2-mediated activation of NF-κB. Sci Rep. 2014; 4:5750. 10.1038/srep05750. 25034888 PMC4102923

[49] Chen H, Yan X, Li Z, Deng Z, Gu J, Zeng F, Li Z, Zhang J. MyD88 orchestrates fatty acid metabolism in tumor-associated macrophages and non-alcoholic fatty liver disease-related hepatocarcinogenesis. Front Immunol. 2025; 16:1589255. 10.3389/fimmu.2025.1589255. 41063983 PMC12500666

[50] Andreuzzi E, Fejza A, Polano M, Poletto E, Camicia L, Carobolante G, Tarticchio G, Todaro F, Di Carlo E, Scarpa M, Scarpa M, Paulitti A, Capuano A, et al. Colorectal cancer development is affected by the ECM molecule EMILIN-2 hinging on macrophage polarization via the TLR-4/MyD88 pathway. J Exp Clin Cancer Res. 2022; 41:60. 10.1186/s13046-022-02271-y. 35148799 PMC8840294

[51] Colonna M. DAP12 signaling: from immune cells to bone modeling and brain myelination. J Clin Invest. 2003; 111:313–14. 10.1172/JCI17745. 12569153 PMC151875

[52] Cioni B, Zaalberg A, van Beijnum JR, Melis MHM, van Burgsteden J, Muraro MJ, Hooijberg E, Peters D, Hofland I, Lubeck Y, de Jong J, Sanders J, Vivié J, et al. Androgen receptor signalling in macrophages promotes TREM-1-mediated prostate cancer cell line migration and invasion. Nat Commun. 2020; 11:4498. 10.1038/s41467-020-18313-y. 32908142 PMC7481219

[53] Visser N, Nelemans LC, He Y, Lourens HJ, Corrales MG, Huls G, Wiersma VR, Schuringa JJ, Bremer E. Signal regulatory protein beta 2 is a novel positive regulator of innate anticancer immunity. Front Immunol. 2023; 14:1287256. 10.3389/fimmu.2023.1287256. 38116002 PMC10729450

[54] Ahmed S, Maratha A, Butt AQ, Shevlin E, Miggin SM. TRIF-mediated TLR3 and TLR4 signaling is negatively regulated by ADAM15. J Immunol. 2013; 190:2217–28. 10.4049/jimmunol.1201630. 23365087

[55] Zheng X, Li S, Yang H. Roles of Toll-Like Receptor 3 in Human Tumors. Front Immunol. 2021; 12:667454. 10.3389/fimmu.2021.667454. 33986756 PMC8111175

[56] Zhao S, Zhang Y, Zhang Q, Wang F, Zhang D. Toll-Like Receptors and Prostate Cancer. Front Immunol. 2014; 5. 10.3389/fimmu.2014.00352. 25101092 PMC4107957

[57] Bali P, Lozano-Pope I, Hernandez J, Estrada MV, Corr M, Turner MA, Bouvet M, Benner C, Obonyo M. TRIF-IFN-I pathway in Helicobacter-induced gastric cancer in an accelerated murine disease model and patient biopsies. iScience. 2024; 27:109457. 10.1016/j.isci.2024.109457. 38558931 PMC10981133

[58] Yuan MM, Xu YY, Chen L, Li XY, Qin J, Shen Y. TLR3 expression correlates with apoptosis, proliferation and angiogenesis in hepatocellular carcinoma and predicts prognosis. BMC Cancer. 2015; 15:245. 10.1186/s12885-015-1262-5. 25884709 PMC4435918

[59] Oshiumi H, Matsumoto M, Funami K, Akazawa T, Seya T. TICAM-1, an adaptor molecule that participates in Toll-like receptor 3–mediated interferon-β induction. Nat Immunol. 2003; 4:161–67. 10.1038/ni886. 12539043

[60] Sarkar SN, Elco CP, Peters KL, Chattopadhyay S, Sen GC. Two Tyrosine Residues of Toll-like Receptor 3 Trigger Different Steps of NF-κB Activation. J Biol Chem. 2007; 282:3423–27. 10.1074/jbc.C600226200. 17178723

[61] Muresan XM, Bouchal J, Culig Z, Souček K. Toll-Like Receptor 3 in Solid Cancer and Therapy Resistance. Cancers. 2020; 12:3227. 10.3390/cancers12113227. 33147700 PMC7692054

[62] Firmal P, Shah VK, Pant R, Chattopadhyay S. RING finger protein TOPORS modulates the expression of tumor suppressor SMAR1 in colorectal cancer via the TLR4-TRIF pathway. Mol Oncol. 2022; 16:1523–40. 10.1002/1878-0261.13126. 34689394 PMC8978522

[63] Kaiser WJ, Offermann MK. Apoptosis Induced by the Toll-Like Receptor Adaptor TRIF Is Dependent on Its Receptor Interacting Protein Homotypic Interaction Motif. J Immunol. 2005; 174:4942–52. 10.4049/jimmunol.174.8.4942. 15814722

[64] Wadhonkar K, Singh Y, Rughetti A, Das S, Yangdol R, Sk MH, Baig MS. Role of cancer cell-derived exosomal glycoproteins in macrophage polarization. Mol Biol Rep. 2025; 52:451. 10.1007/s11033-025-10535-x. 40347313

[65] Wadhonkar K, Das S, Subramanian R, Sk MH, Singh Y, Baig MS. The effect of cancer cell-derived exosomal proteins on macrophage polarization: An in-depth review. Exp Cell Res. 2025; 444:114393. 10.1016/j.yexcr.2024.114393. 39710293

[66] Malagrinò F, Puglisi E, Pagano L, Travaglini-Allocatelli C, Toto A. GRB2: A dynamic adaptor protein orchestrating cellular signaling in health and disease. Biochem Biophys Rep. 2024; 39:101803. 10.1016/j.bbrep.2024.101803. 39175664 PMC11340617

[67] Shen Q, Wang S, Wu K, Wang L, Gong W, Lu G, Chen W, Yuan C, Tu B, Li W, Wang Y, Yang W. Identification of Grb2 protein as a potential mediator of macrophage activation in acute pancreatitis based on bioinformatics and experimental verification. Front Immunol. 2025; 16:1575880. 10.3389/fimmu.2025.1575880. 40491924 PMC12146204

[68] Mao H, Zhao X, Sun S. NF-κB in inflammation and cancer. Cell Mol Immunol. 2025; 22:811–39. 10.1038/s41423-025-01310-w. 40562870 PMC12310982

[69] Li C, Xu X, Wei S, Jiang P, Xue L, Wang J. Tumor-associated macrophages: potential therapeutic strategies and future prospects in cancer. J Immunother Cancer. 2021; 9:e001341. 10.1136/jitc-2020-001341. 33504575 PMC8728363

[70] Kimura T, Nada S, Takegahara N, Okuno T, Nojima S, Kang S, Ito D, Morimoto K, Hosokawa T, Hayama Y, Mitsui Y, Sakurai N, Sarashina-Kida H, et al. Polarization of M2 macrophages requires Lamtor1 that integrates cytokine and amino-acid signals. Nat Commun. 2016; 7:13130. 10.1038/ncomms13130. 27731330 PMC5064021

[71] Li J, Liu N, Tang L, Yan B, Chen X, Zhang J, Peng C. The relationship between TRAF6 and tumors. Cancer Cell Int. 2020; 20:429. 10.1186/s12935-020-01517-z. 32905356 PMC7469280

[72] Dan H, Liu S, Liu J, Liu D, Yin F, Wei Z, Wang J, Zhou Y, Jiang L, Ji N, Zeng X, Li J, Chen Q. RACK1 promotes cancer progression by increasing the M2/M1 macrophage ratio via the NF-κB pathway in oral squamous cell carcinoma. Mol Oncol. 2020; 14:795–807. 10.1002/1878-0261.12644. 31997535 PMC7138402

[73] Yang X, Ji C, Qi Y, Huang J, Hu L, Zhou Y, Zou L, Xia Y, Tan F, Yao Y, Chen D. Signal-transducing adaptor protein 1 (STAP1) in microglia promotes the malignant progression of glioma. J Neurooncol. 2023; 164:127–39. 10.1007/s11060-023-04390-8. 37462801 PMC10462508

[74] Liu ZZ, Han ZD, Liang YK, Chen JX, Wan S, Zhuo YJ, Cai ZD, Deng YL, Lin ZY, Mo RJ, He HC, Zhong WD. TRIB1 induces macrophages to M2 phenotype by inhibiting IKB-zeta in prostate cancer. Cell Signal. 2019; 59:152–62. 10.1016/j.cellsig.2019.03.017. 30926388

[75] Willems M, Dubois N, Musumeci L, Bours V, Robe PA. IκBζ: an emerging player in cancer. Oncotarget. 2016; 7:66310–22. 10.18632/oncotarget.11624. 27579619 PMC5323236

[76] Ding CB, Yu WN, Feng JH, Luo JM. Structure and function of Gab2 and its role in cancer (Review). Mol Med Rep. 2015; 12:4007–14. 10.3892/mmr.2015.3951. 26095858 PMC4526075

[77] Ding C, Luo J, Fan X, Li L, Li S, Wen K, Feng J, Wu G. Elevated Gab2 induces tumor growth and angiogenesis in colorectal cancer through upregulating VEGF levels. J Exp Clin Cancer Res. 2017; 36:56. 10.1186/s13046-017-0524-2. 28420432 PMC5395829

[78] Wöhrle FU, Halbach S, Aumann K, Schwemmers S, Braun S, Auberger P, Schramek D, Penninger JM, Laßmann S, Werner M, Waller CF, Pahl HL, Zeiser R, et al. Gab2 signaling in chronic myeloid leukemia cells confers resistance to multiple Bcr-Abl inhibitors. Leukemia. 2013; 27:118–29. 10.1038/leu.2012.222. 22858987

[79] Ke Y, Wu D, Princen F, Nguyen T, Pang Y, Lesperance J, Muller WJ, Oshima RG, Feng GS. Role of Gab2 in mammary tumorigenesis and metastasis. Oncogene. 2007; 26:4951–60. 10.1038/sj.onc.1210315. 17310989

[80] Wang Y, Sheng Q, Spillman MA, Behbakht K, Gu H. Gab2 regulates the migratory behaviors and E-cadherin expression via activation of the PI3K pathway in ovarian cancer cells. Oncogene. 2012; 31:2512–20. 10.1038/onc.2011.435. 21996746 PMC3262088

[81] Kondreddy V, Magisetty J, Keshava S, Rao LVM, Pendurthi UR. Gab2 (Grb2-Associated Binder2) Plays a Crucial Role in Inflammatory Signaling and Endothelial Dysfunction. Arterioscler Thromb Vasc Biol. 2021; 41:1987–2005. 10.1161/ATVBAHA.121.316153. 33827252 PMC8147699

[82] Wu K, Lin K, Li X, Yuan X, Xu P, Ni P, Xu D. Redefining Tumor-Associated Macrophage Subpopulations and Functions in the Tumor Microenvironment. Front Immunol. 2020; 11:1731. 10.3389/fimmu.2020.01731. 32849616 PMC7417513

[83] Gao X, Long R, Qin M, Zhu W, Wei L, Dong P, Chen J, Luo J, Feng J. Gab2 promotes the growth of colorectal cancer by regulating the M2 polarization of tumor-associated macrophages. Int J Mol Med. 2023; 53:3. 10.3892/ijmm.2023.5327. 37937666 PMC10688767

[84] Rajpoot S, Wary KK, Ibbott R, Liu D, Saqib U, Thurston TLM, Baig MS. TIRAP in the Mechanism of Inflammation. Front Immunol. 2021; 12:697588. 10.3389/fimmu.2021.697588. 34305934 PMC8297548

[85] Fitzgerald KA, Palsson-McDermott EM, Bowie AG, Jefferies CA, Mansell AS, Brady G, Brint E, Dunne A, Gray P, Harte MT, McMurray D, Smith DE, Sims JE, et al. Mal (MyD88-adapter-like) is required for Toll-like receptor-4 signal transduction. Nature. 2001; 413:78–83. 10.1038/35092578. 11544529

[86] Hu X, Fatima S, Chen M, Xu K, Huang C, Gong RH, Su T, Wong HLX, Bian Z, Kwan HY. Toll-like receptor 4 is a master regulator for colorectal cancer growth under high-fat diet by programming cancer metabolism. Cell Death Dis. 2021; 12:791. 10.1038/s41419-021-04076-x. 34385421 PMC8360949

[87] Hao S, Li S, Wang J, Yan Y, Ai X, Zhang J, Ren Y, Wu T, Liu L, Wang C. Phycocyanin Exerts Anti-Proliferative Effects through Down-Regulating TIRAP/NF-κB Activity in Human Non-Small Cell Lung Cancer Cells. Cells. 2019; 8:588. 10.3390/cells8060588. 31207932 PMC6627414

[88] Xu D, Ji Z, Qiang L. Molecular Characteristics, Clinical Implication, and Cancer Immunity Interactions of Pyroptosis-Related Genes in Breast Cancer. Front Med (Lausanne). 2021; 8:702638. 10.3389/fmed.2021.702638. 34589498 PMC8473741

[89] Chen Y, Zhou P, Deng Y, Cai X, Sun M, Sun Y, Wu D. ALKBH5-mediated m6 A demethylation of TIRAP mRNA promotes radiation-induced liver fibrosis and decreases radiosensitivity of hepatocellular carcinoma. Clin Transl Med. 2023; 13:e1198. 10.1002/ctm2.1198. 36792369 PMC9931500

[90] Rajpoot S, Kumar A, Zhang KYJ, Gan SH, Baig MS. TIRAP-mediated activation of p38 MAPK in inflammatory signaling. Sci Rep. 2022; 12:5601. 10.1038/s41598-022-09528-8. 35379857 PMC8979995

[91] Hernández-Varas P, Coló GP, Bartolomé RA, Paterson A, Medraño-Fernández I, Arellano-Sánchez N, Cabañas C, Sánchez-Mateos P, Lafuente EM, Boussiotis VA, Strömblad S, Teixidó J. Rap1-GTP-interacting Adaptor Molecule (RIAM) Protein Controls Invasion and Growth of Melanoma Cells. J Biol Chem. 2011; 286:18492–504. 10.1074/jbc.M110.189811. 21454517 PMC3099666

[92] Lafuente EM, van Puijenbroek AA, Krause M, Carman CV, Freeman GJ, Berezovskaya A, Constantine E, Springer TA, Gertler FB, Boussiotis VA. RIAM, an Ena/VASP and Profilin ligand, interacts with Rap1-GTP and mediates Rap1-induced adhesion. Dev Cell. 2004; 7:585–95. 10.1016/j.devcel.2004.07.021. 15469846

[93] Sari-Ak D, Torres-Gomez A, Yazicioglu YF, Christofides A, Patsoukis N, Lafuente EM, Boussiotis VA. Structural, biochemical, and functional properties of the Rap1-Interacting Adaptor Molecule (RIAM). Biomed J. 2022; 45:289–98. 10.1016/j.bj.2021.09.005. 34601137 PMC9250098

[94] Patsoukis N, Bardhan K, Weaver JD, Sari D, Torres-Gomez A, Li L, Strauss L, Lafuente EM, Boussiotis VA. The adaptor molecule RIAM integrates signaling events critical for integrin-mediated control of immune function and cancer progression. Sci Signal. 2017; 10:eaam8298. 10.1126/scisignal.aam8298. 28831022

[95] Christofides A, Cao C, Pal R, Boussiotis VA. RIAM regulates myeloid cell fate commitment and macrophage polarization and controls tumor progression. J Immunol. 2021; 206:101.04. 10.4049/jimmunol.206.Supp.101.04.33288546

[96] Nakatani T, Tsujimoto K, Park J, Jo T, Kimura T, Hayama Y, Konaka H, Morita T, Kato Y, Nishide M, Koyama S, Nada S, Okada M, et al. The lysosomal Ragulator complex plays an essential role in leukocyte trafficking by activating myosin II. Nat Commun. 2021; 12:3333. 10.1038/s41467-021-23654-3. 34099704 PMC8184920

[97] Malek M, Guillaumot P, Huber AL, Lebeau J, Pétrilli V, Kfoury A, Mikaelian I, Renno T, Manié SN. LAMTOR1 depletion induces p53-dependent apoptosis via aberrant lysosomal activation. Cell Death Dis. 2012; 3:e300. 10.1038/cddis.2012.39. 22513874 PMC3358017

[98] Hosokawa T, Kimura T, Nada S, Okuno T, Ito D, Kang S, Nojima S, Yamashita K, Nakatani T, Hayama Y, Kato Y, Kinehara Y, Nishide M, et al. Lamtor1 Is Critically Required for CD4^+^ T Cell Proliferation and Regulatory T Cell Suppressive Function. J Immunol. 2017; 199:2008–19. 10.4049/jimmunol.1700157. 28768723

[99] Sun Y, Guan Z, Sheng Q, Duan W, Zhao H, Zhou J, Deng Q, Pei X. N-myristoyltransferase-1 deficiency blocks myristoylation of LAMTOR1 and inhibits bladder cancer progression. Cancer Lett. 2022; 529:126–38. 10.1016/j.canlet.2022.01.001. 34999170

[100] Wu B, Huang X, Shi X, Jiang M, Liu H, Zhao L. LAMTOR1 decreased exosomal PD-L1 to enhance immunotherapy efficacy in non-small cell lung cancer. Mol Cancer. 2024; 23:184. 10.1186/s12943-024-02099-4. 39223601 PMC11367890

[101] Zhao L, Gao N, Peng X, Chen L, Meng T, Jiang C, Jin J, Zhang J, Duan Q, Tian H, Weng L, Wang X, Tan X, et al. TRAF4-Mediated LAMTOR1 Ubiquitination Promotes mTORC1 Activation and Inhibits the Inflammation-Induced Colorectal Cancer Progression. Adv Sci (Weinh). 2024; 11:e2301164. 10.1002/advs.202301164. 38229144 PMC10966530

[102] Soave DF, Miguel MP, Tomé FD, de Menezes LB, Nagib PR, Celes MR. The Fate of the Tumor in the Hands of Microenvironment: Role of TAMs and mTOR Pathway. Mediators Inflamm. 2016; 2016:8910520. 10.1155/2016/8910520. 28074082 PMC5198177

[103] Arch RH, Gedrich RW, Thompson CB. Tumor necrosis factor receptor-associated factors (TRAFs)--a family of adapter proteins that regulates life and death. Genes Dev. 1998; 12:2821–30. 10.1101/gad.12.18.2821. 9744859

[104] Wu L, Chen X, Zhao J, Martin B, Zepp JA, Ko JS, Gu C, Cai G, Ouyang W, Sen G, Stark GR, Su B, Vines CM, et al. A novel IL-17 signaling pathway controlling keratinocyte proliferation and tumorigenesis via the TRAF4–ERK5 axis. J Exp Med. 2015; 212:1571–87. 10.1084/jem.20150204. 26347473 PMC4577838

[105] Yamamoto H, Ryu J, Min E, Oi N, Bai R, Zykova TA, Yu DH, Moriyama K, Bode AM, Dong Z. TRAF1 Is Critical for DMBA/Solar UVR-Induced Skin Carcinogenesis. J Invest Dermatol. 2017; 137:1322–32. 10.1016/j.jid.2016.12.026. 28131816 PMC5995119

[106] Wang Q, Gao G, Zhang T, Yao K, Chen H, Park MH, Yamamoto H, Wang K, Ma W, Malakhova M, Bode AM, Dong Z. TRAF1 Is Critical for Regulating the BRAF/MEK/ERK Pathway in Non–Small Cell Lung Carcinogenesis. Cancer Res. 2018; 78:3982–94. 10.1158/0008-5472.CAN-18-0429. 29748372 PMC6050072

[107] Peramuhendige P, Marino S, Bishop RT, de Ridder D, Khogeer A, Baldini I, Capulli M, Rucci N, Idris AI. TRAF2 in osteotropic breast cancer cells enhances skeletal tumour growth and promotes osteolysis. Sci Rep. 2018; 8:39. 10.1038/s41598-017-18327-5. 29311633 PMC5758572

[108] Zhao J, Li H, Min L, Han X, Shu P, Yang Y, Gan Q, Wang X, Wang H, Ruan Y, Qin J, Sun Y, Qin X. High expression of tumor necrosis factor receptor-associated factor 2 promotes tumor metastasis and is associated with unfavorable prognosis in gastric cancer. J Gastroenterol Hepatol. 2018; 33:431–42. 10.1111/jgh.13818. 28482378

[109] Liang Z, Li X, Liu S, Li C, Wang X, Xing J. MiR-141-3p inhibits cell proliferation, migration and invasion by targeting TRAF5 in colorectal cancer. Biochem Biophys Res Commun. 2019; 514:699–705. 10.1016/j.bbrc.2019.05.002. 31078266

[110] Tan HY, Wang N, Man K, Tsao SW, Che CM, Feng Y. Autophagy-induced RelB/p52 activation mediates tumour-associated macrophage repolarisation and suppression of hepatocellular carcinoma by natural compound baicalin. Cell Death Dis. 2015; 6:e1942. 10.1038/cddis.2015.271. 26492375 PMC4632300

[111] Glaus Garzon JF, Pastrello C, Jurisica I, Hottiger MO, Wenger RH, Borsig L. Tumor cell endogenous HIF-1α activity induces aberrant angiogenesis and interacts with TRAF6 pathway required for colorectal cancer development. Neoplasia. 2020; 22:745–58. 10.1016/j.neo.2020.10.006. 33142239 PMC7588814

[112] Jin J, Xiao Y, Hu H, Zou Q, Li Y, Gao Y, Ge W, Cheng X, Sun SC. Proinflammatory TLR signalling is regulated by a TRAF2-dependent proteolysis mechanism in macrophages. Nat Commun. 2015; 6:5930. 10.1038/ncomms6930. 25565375 PMC4286812

[113] Liu X, Jiang B, Hao H, Liu Z. CARD9 Signaling, Inflammation, and Diseases. Front Immunol. 2022; 13:880879. 10.3389/fimmu.2022.880879. 35432375 PMC9005907

[114] Zhong X, Chen B, Yang L, Yang Z. Molecular and physiological roles of the adaptor protein CARD9 in immunity. Cell Death Dis. 2018; 9:52. 10.1038/s41419-017-0084-6. 29352133 PMC5833731

[115] Miwa N, Nagano T, Jimbo N, Dokuni R, Kiriu T, Mimura C, Yasuda Y, Katsurada M, Yamamoto M, Tachihara M, Tanaka Y, Kobayashi K, Itoh T, et al. Caspase Recruitment Domain-Containing Protein 9 Expression is a Novel Prognostic Factor for Lung Adenocarcinoma. Onco Targets Ther. 2020; 13:9005–13. 10.2147/OTT.S265539. 32982291 PMC7498929

[116] Wang Y, Wang C, Zhu Y. CARD9 contributes to ovarian cancer cell proliferation, cycle arrest, and cisplatin sensitivity. BMC Mol Cell Biol. 2022; 23:49. 10.1186/s12860-022-00447-0. 36443670 PMC9703781

[117] Luo P, Ming Z, Yang Z. A Critical Role for CARD9 in Intestinal Microbiota Modulation and Colorectal Malignancies. Front Biosci (Landmark Ed). 2022; 27:320. 10.31083/j.fbl2712320. 36624940

[118] Bergmann H, Roth S, Pechloff K, Kiss EA, Kuhn S, Heikenwälder M, Diefenbach A, Greten FR, Ruland J. Card9-dependent IL-1β regulates IL-22 production from group 3 innate lymphoid cells and promotes colitis-associated cancer. Eur J Immunol. 2017; 47:1342–53. 10.1002/eji.201646765. 28586167 PMC5600091

[119] Matsuda T, Oritani K. Possible Therapeutic Applications of Targeting STAP Proteins in Cancer. Biol Pharm Bull. 2021; 44:1810–18. 10.1248/bpb.b21-00672. 34853263

[120] Toda J, Ichii M, Oritani K, Shibayama H, Tanimura A, Saito H, Yokota T, Motooka D, Okuzaki D, Kitai Y, Muromoto R, Kashiwakura JI, Matsuda T, et al. Signal-transducing adapter protein-1 is required for maintenance of leukemic stem cells in CML. Oncogene. 2020; 39:5601–15. 10.1038/s41388-020-01387-9. 32661325 PMC7441008

[121] Matsuda T, Oritani K. STAP-2 Adaptor Protein Regulates Multiple Steps of Immune and Inflammatory Responses. Biol Pharm Bull. 2021; 44:895–901. 10.1248/bpb.b21-00224. 34193686

[122] Ikeda O, Mizushima A, Sekine Y, Yamamoto C, Muromoto R, Nanbo A, Oritani K, Yoshimura A, Matsuda T. Involvement of STAP-2 in Brk-mediated phosphorylation and activation of STAT5 in breast cancer cells. Cancer Sci. 2011; 102:756–61. 10.1111/j.1349-7006.2010.01842.x. 21205088

[123] Kitai Y, Iwakami M, Saitoh K, Togi S, Isayama S, Sekine Y, Muromoto R, Kashiwakura J, Yoshimura A, Oritani K, Matsuda T. STAP-2 protein promotes prostate cancer growth by enhancing epidermal growth factor receptor stabilization. J Biol Chem. 2017; 292:19392–99. 10.1074/jbc.M117.802884. 28986450 PMC5702677

[124] Sekine Y. Adaptor Protein STAP-2 Modulates Cellular Signaling in Immune Systems. Biol Pharm Bull. 2014; 37:185–94. 10.1248/bpb.b13-00421. 24492713

[125] Sekine Y, Yumioka T, Yamamoto T, Muromoto R, Imoto S, Sugiyma K, Oritani K, Shimoda K, Minoguchi M, Akira S, Yoshimura A, Matsuda T. Modulation of TLR4 signaling by a novel adaptor protein signal-transducing adaptor protein-2 in macrophages. J Immunol. 2006; 176:380–89. 10.4049/jimmunol.176.1.380. 16365431

[126] Tian R, Tian J, Zuo X, Ren S, Zhang H, Liu H, Wang Z, Cui Y, Niu R, Zhang F. RACK1 facilitates breast cancer progression by competitively inhibiting the binding of β-catenin to PSMD2 and enhancing the stability of β-catenin. Cell Death Dis. 2023; 14:685. 10.1038/s41419-023-06191-3. 37848434 PMC10582012

[127] Li J, Guo Y, Feng X, Wang Z, Wang Y, Deng P, Zhang D, Wang R, Xie L, Xu X, Zhou Y, Ji N, Hu J, et al. Receptor for activated C kinase 1 (RACK1): a regulator for migration and invasion in oral squamous cell carcinoma cells. J Cancer Res Clin Oncol. 2012; 138:563–71. 10.1007/s00432-011-1097-7. 22207523 PMC11824729

[128] Berns H, Humar R, Hengerer B, Kiefer FN, Battegay EJ. RACK1 is up-regulated in angiogenesis and human carcinomas. FASEB J. 2000; 14:2549–58. 10.1096/fj.99-1038com. 11099474

[129] Li G, Ji XD, Gao H, Zhao JS, Xu JF, Sun ZJ, Deng YZ, Shi S, Feng YX, Zhu YQ, Wang T, Li JJ, Xie D. EphB3 suppresses non-small-cell lung cancer metastasis via a PP2A/RACK1/Akt signaling complex. Nat Commun. 2012; 3:667. 10.1038/ncomms1675. 22314363

[130] Xiao T, Zhu W, Huang W, Lu SS, Li XH, Xiao ZQ, Yi H. RACK1 promotes tumorigenicity of colon cancer by inducing cell autophagy. Cell Death Dis. 2018; 9:1148. 10.1038/s41419-018-1113-9. 30451832 PMC6242835

[131] Shi S, Deng YZ, Zhao JS, Ji XD, Shi J, Feng YX, Li G, Li JJ, Zhu D, Koeffler HP, Zhao Y, Xie D. RACK1 Promotes Non-small-cell Lung Cancer Tumorigenicity through Activating Sonic Hedgehog Signaling Pathway. J Biol Chem. 2012; 287:7845–58. 10.1074/jbc.M111.315416. 22262830 PMC3318742

[132] Wang Z, Zhang B, Jiang L, Zeng X, Chen Y, Feng X, Guo Y, Chen Q. RACK1, an excellent predictor for poor clinical outcome in oral squamous carcinoma, similar to Ki67. Eur J Cancer. 2009; 45:490–96. 10.1016/j.ejca.2008.11.012. 19087901

[133] Nagashio R, Sato Y, Matsumoto T, Kageyama T, Satoh Y, Shinichiro R, Masuda N, Goshima N, Jiang SX, Okayasu I. Expression of RACK1 is a novel biomarker in pulmonary adenocarcinomas. Lung Cancer. 2010; 69:54–59. 10.1016/j.lungcan.2009.09.015. 19892429

[134] Cao X, Xu J, Liu X, Xu J, Wang W, Li Q, Chen Q, Xu Z, Liu X. RACK1: A superior independent predictor for poor clinical outcome in breast cancer. Int J Cancer. 2010; 127:1172–79. 10.1002/ijc.25120. 20020495

[135] Yokoyama T, Nakamura T. Tribbles in disease: Signaling pathways important for cellular function and neoplastic transformation. Cancer Sci. 2011; 102:1115–22. 10.1111/j.1349-7006.2011.01914.x. 21338441

[136] Niespolo C, Johnston JM, Deshmukh SR, Satam S, Shologu Z, Villacanas O, Sudbery IM, Wilson HL, Kiss-Toth E. Tribbles-1 Expression and Its Function to Control Inflammatory Cytokines, Including Interleukin-8 Levels are Regulated by miRNAs in Macrophages and Prostate Cancer Cells. Front Immunol. 2020; 11:574046. 10.3389/fimmu.2020.574046. 33329538 PMC7728618

[137] Chavey C, Mühlbauer M, Bossard C, Freund A, Durand S, Jorgensen C, Jobin C, Lazennec G. Interleukin-8 expression is regulated by histone deacetylases through the nuclear factor-kappaB pathway in breast cancer. Mol Pharmacol. 2008; 74:1359–66. 10.1124/mol.108.047332. 18669446

[138] Waugh DJ, Wilson C. The interleukin-8 pathway in cancer. Clin Cancer Res. 2008; 14:6735–41. 10.1158/1078-0432.CCR-07-4843. 18980965

[139] Kim T, Johnston J, Castillo-Lluva S, Cimas FJ, Hamby S, and Cardiogenics Consortium*. TRIB1 regulates tumor growth via controlling tumor-associated macrophage phenotypes and is associated with breast cancer survival and treatment response. Theranostics. 2022; 12:3584–600. 10.7150/thno.72192. 35664073 PMC9131267

[140] Wang L, Hensley CR, Howell ME, Ning S. Bioinformatics-Driven Identification of p62 as A Crucial Oncogene in Liver Cancer. Front Oncol. 2022; 12:923009. 10.3389/fonc.2022.923009. 35814476 PMC9263135

[141] Mao Y, Deng SJ, Su YJ, Diao C, Peng Y, Ma JF, Cheng RC. The role of P62 in the development of human thyroid cancer and its possible mechanism. Cancer Genet. 2021; 256-257:5–16. 10.1016/j.cancergen.2021.02.008. 33780725

[142] Hennig P, Fenini G, Di Filippo M, Karakaya T, Beer HD. The Pathways Underlying the Multiple Roles of p62 in Inflammation and Cancer. Biomedicines. 2021; 9:707. 10.3390/biomedicines9070707. 34206503 PMC8301319

[143] Zhang X, Dai M, Li S, Li M, Cheng B, Ma T, Zhou Z. The emerging potential role of p62 in cancer treatment by regulating metabolism. Trends Endocrinol Metab. 2023; 34:474–88. 10.1016/j.tem.2023.05.004. 37349161

[144] Xu Y, Liao C, Liu R, Liu J, Chen Z, Zhao H, Li Z, Chen L, Wu C, Tan H, Liu W, Li W. IRGM promotes glioma M2 macrophage polarization through p62/TRAF6/NF-κB pathway mediated IL-8 production. Cell Biol Int. 2019; 43:125–35. 10.1002/cbin.11061. 30288851 PMC13397409

[145] Yang W, Su G, Liu Y. Silencing p62 reduces ox-LDL-induced M1 polarization and inflammation in macrophages by inhibiting mTOR/NF-κB signaling pathways. Eur J Inflamm. 2022; 20:1721727X221110348. 10.1177/1721727X221110348.

[146] Shi JH, Liu LN, Song DD, Liu WW, Ling C, Wu FX, Wang TT, Liu B, Cui NP, Qin Y, Ni ZY. TRAF3/STAT6 axis regulates macrophage polarization and tumor progression. Cell Death Diff. 2023; 30:2005–16. 10.1038/s41418-023-01194-1. 37474750 PMC10406838

[147] Raizada S, Obukhov AG, Bharti S, Wadhonkar K, Baig MS. Pharmacological targeting of adaptor proteins in chronic inflammation. Inflamm Res. 2024; 73:1645–56. 10.1007/s00011-024-01921-5. 39052063

[148] Mikolajczyk A, Mitula F, Popiel D, Kaminska B, Wieczorek M, Pieczykolan J. Two-Front War on Cancer-Targeting TAM Receptors in Solid Tumour Therapy. Cancers (Basel). 2022; 14:2488. 10.3390/cancers14102488. 35626092 PMC9140196

[149] Maemoto T, Sasaki Y, Okuyama F, Kitai Y, Oritani K, Matsuda T. Potential of targeting signal-transducing adaptor protein-2 in cancer therapeutic applications. Explor Target Anti-Tumor Ther. 2024; 5. 10.37349/etat.2024.00216. PMC11090684 38745775

[150] Xie L, Jiang FC, Zhang LM, He WT, Liu JH, Li MQ, Zhang X, Xing S, Guo H, Zhou P. Targeting of MyD88 Homodimerization by Novel Synthetic Inhibitor TJ-M2010-5 in Preventing Colitis-Associated Colorectal Cancer. J Natl Cancer Inst. 2016; 108:djv364. 10.1093/jnci/djv364. 26712311

[151] Lu S, He T, Zhang Y, Zhou B, Zhang Q, Yan S. The MyD88 inhibitor, ST2825, induces cell cycle arrest and apoptosis by suppressing the activation of the NF-κB/AKT1/p21 pathway in pancreatic cancer. Oncol Rep. 2023; 50:148. 10.3892/or.2023.8585. 37326109 PMC10308493

[152] Witort E, Lulli M, Carloni V, Capaccioli S. Anticancer activity of an antisense oligonucleotide targeting TRADD combined with proteasome inhibitors in chemoresistant hepatocellular carcinoma cells. J Chemother. 2013; 25:292–97. 10.1179/1973947813Y.0000000087. 24070137

[153] Su P, Li O, Ke K, Jiang Z, Wu J, Wang Y, Mou Y, Jin W. Targeting tumor-associated macrophages: Critical players in tumor progression and therapeutic strategies (Review). Int J Oncol. 2024; 64:60. 10.3892/ijo.2024.5648. 38695252 PMC11087038

[154] Li D, Rudloff U. Emerging therapeutics targeting tumor-associated macrophages for the treatment of solid organ cancers. Expert Opin Emerg Drugs. 2025; 30:109–47. 10.1080/14728214.2025.2504376. 40353504 PMC12232465

[155] Borowicz P, Chan H, Hauge A, Spurkland A. Adaptor proteins: Flexible and dynamic modulators of immune cell signaling. Scand J Immunol. 2020; 92:e12951. 10.1111/sji.12951. 32734639

[156] Luo LY, Hahn WC. Oncogenic Signaling Adaptor Proteins. J Genet Genomics. 2015; 42:521–29. 10.1016/j.jgg.2015.09.001. 26554907 PMC4643408

[157] Valdes-Mora F, Handler K, Law AMK, Salomon R, Oakes SR, Ormandy CJ, Gallego-Ortega D. Single-Cell Transcriptomics in Cancer Immunobiology: The Future of Precision Oncology. Front Immunol. 2018; 9:2582. 10.3389/fimmu.2018.02582. 30483257 PMC6240655

[158] Kelm JM, Pandey DS, Malin E, Kansou H, Arora S, Kumar R, Gavande NS. PROTAC’ing oncoproteins: targeted protein degradation for cancer therapy. Mol Cancer. 2023; 22:62. 10.1186/s12943-022-01707-5. 36991452 PMC10061819

[159] Drewniak-Świtalska M, Fortuna P, Krzystek-Korpacka M. Negative Immune Checkpoint Inhibitors. Pharmaceutics. 2025; 17:713. 10.3390/pharmaceutics17060713. 40574024 PMC12195700

[160] Motevasseli M, Darvishi M, Khoshnevisan A, Zeinalizadeh M, Saffar H, Bayat S, Najafi A, Abbaspour MJ, Mamivand A, Olson SB, Tabrizi M. Distinct tumor-TAM interactions in IDH-stratified glioma microenvironments unveiled by single-cell and spatial transcriptomics. Acta Neuropathol Commun. 2024; 12:133. 10.1186/s40478-024-01837-5. 39148129 PMC11328419

[161] Atre R, Sharma R, Vadim G, Solanki K, Wadhonkar K, Singh N, Patidar P, Khabiya R, Samaur H, Banerjee S, Baig MS. The indispensability of macrophage adaptor proteins in chronic inflammatory diseases. Int Immunopharmacol. 2023; 119:110176. 10.1016/j.intimp.2023.110176. 37104916

[162] Hofstatter Azambuja J, Yerneni S, Maurer L, Crentsil H, Debom G, Klei L, Smyers M, Sneiderman C, Schwab K, Acharya R, Wu YL, Ekambaram P, Hu D, et al. TMIC-03. Card9 blockade offers a new opportunity to reprogram tumor-associated macrophages in glioblastoma. Neuro-Oncol. 2024; 26:viii297. 10.1093/neuonc/noae165.1181.

